# Supplementary material for: The interactome of intact mitochondria by cross-linking mass spectrometry provides evidence for coexisting respiratory supercomplexes
Source: Mol Cell Proteomics. 2017 Dec 8;17(2):216–32. doi: 10.1074/mcp.RA117.000470 (PMC5795388; doi:10.1074/mcp.RA117.000470)
Supplement: Supplemental Data [file supp_RA117.000470_133922_0_supp_23978_fzffwf.zip › spectra_annotation/mito_spectra_annotation/77-1-3-1-3-1.pdf]

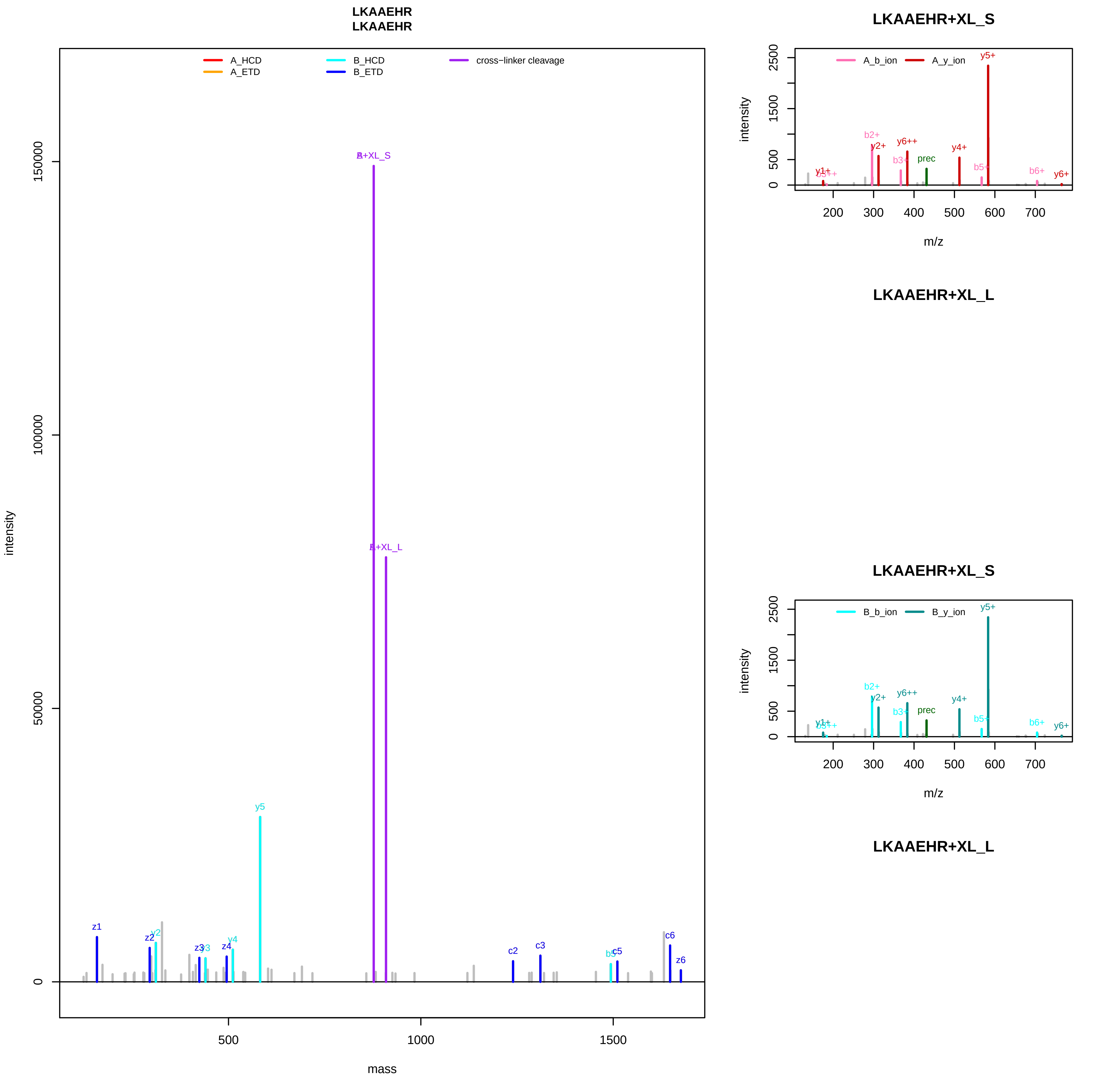
LKAAEHR
LKAAEHR
A_HCD
A_ETD
B_HCD
B_ETD
cross−linker cleavage
B+XL_S
B+XL_L
intensity
mass
0
50000
100000
150000
500
1000
1500
z1
z2
y2
z3
y3
z4
y4
y5
c2
c3
b5
c5
c6
z6
LKAAEHR+XL_S
A_b_ion
A_y_ion
intensity
m/z
y1+
b3++
b2+
y2+
b3+
y6++
prec
y4+
y5+
b5+
b6+
y6+
LKAAEHR+XL_L
LKAAEHR+XL_S
B_b_ion
B_y_ion
intensity
m/z
y1+
b3++
b2+
y2+
b3+
y6++
prec
y4+
y5+
b5+
b6+
y6+
LKAAEHR+XL_L
